# Behavioral and social determinants of early childhood caries among Palestinian preschoolers in Jerusalem area: a cross-sectional study

**DOI:** 10.1186/s12903-023-02809-2

**Published:** 2023-03-15

**Authors:** Elham Kateeb, Sungwoo Lim, Saif Amer, Amid Ismail

**Affiliations:** 1 Oral Health Research and Promotion Unit, Al-Quds University, Jerusalem, Palestine; 2 Kornberg School of Dentistry, Temple University, Philadelphia, PA USA; 3 Horizon Academy, Al-Nayzak, Ramallah, Palestine

**Keywords:** Early childhood caries, Social determinants, Preschoolers, Locus of control, Social support, Parental stress

## Abstract

**Background:**

This study came to determine the prevalence of Early Childhood Carries (ECC) among preschoolers in a marginalized population and describe the influence of behavioral and social determinants on the development of ECC.

**Methods:**

This is a cross-sectional study that was carried out in four random preschools in the Jerusalem Governorate of the Occupied Palestinian Territories. All children aged 3–5 years old in the selected schools were screened for ECC using the decayed, missing, and filled teeth index (dmft). Data on children’s socio-economic, feeding habits, hygiene habits, access to care, parental level of stress, social support, and locus of control were collected by a validated questionnaire sent to the children’s main caregivers. Descriptive statistics were generated and bivariable and multivariable analyses were used to explain the influence of different behavioral and social determinants on ECC levels.

**Results:**

Four hundred and fifty-seven preschoolers completed the questionnaire and the clinical screening. Ninety-seven percent (n = 447) had experienced dental decay, with an average dmft score of 6.6 ± 4.3. After accounting for potential confounding, parents’ internal locus of control was associated with lower dental caries among children (IRR = 0.97, 95% CI = 0.97, 0.98). Having routine, preventive visits versus never seeing a dentist were associated with lower dmft scores (IRR = 0.42, 95% CI = 0.33, 0.52). Night feeding habits (putting things other than water in the baby bottle at night, having children sleep while being breastfed at night) were positively associated with children’s dental caries (IRR = 1.06, 95% CI = 1.04, 1.09: IRR = 1.15, 95% CI = 1.03, 1.29, respectively). Not adding sugar to the bottle was negatively associated with children’s dental caries (IRR = 0.86, 95% CI = 0.74, 1.00).

**Conclusions:**

Preschoolers in this study suffered from high dental caries experience. Although infant feeding habits were key factors in explaining the elevated level of the disease, system and socio-psychological factors were also detrimental to ECC prevalence. Policies and interventions to alleviate the burden of ECC need to address socioeconomic determinants of health in addition to feeding and hygiene practices.

**Supplementary Information:**

The online version contains supplementary material available at 10.1186/s12903-023-02809-2.

## Introduction

According to the Global Burden of Disease Study, in 2017 [1], more than 530 million children have experienced dental caries in their primary teeth making Early Childhood Caries (ECC) one of the most prevalent childhood diseases and serious public health problems. ECC is defined as the presence of one or more decayed, missing, or filled tooth surfaces (dmfs) in any primary tooth of children under 71 months old [2]. Although preventable, children around the world, especially those from socially and economically disadvantaged backgrounds suffer from the detrimental consequence of dental caries [3]. Health complications of ECC include pain, infection, total destruction of teeth, difficulty in chewing foods resulting in digestive disorders, as well as psychological and social consequences that lead to diminished health-related quality of life [4, 5].

ECC is a microbiome-mediated, sugar-driven, multifactorial, dynamic disease that results in the imbalance of demineralization and remineralization of dental hard tissues [6].

Environmental and cultural factors play a key role in shaping dietary habits and personal preventive practices that affect ECC [7]. It is documented in the literature that Middle Eastern countries suffer from high sugar consumption [8–10]. In Palestine, unfavorable dietary habits such as high consumption of added sugars, sweets, soda, and energy drinks were found to be related to higher rates of dental caries in older children [11].

In addition to biological and behavioral factors, ECC is influenced by socio-psychological, and economic factors predisposed by children’s environment, which is known by social determinants of health [12].

Mothers’ knowledge, beliefs, and practices related to oral health and dental care services are major influencers on children's oral health practices and the development of ECC [13, 14] 14, 15. A previous survey of Palestinian mothers in Jerusalem Governorate found a strong negative association between ECC and the mothers’ oral health literacy and their beliefs about receiving dental care during pregnancy and elevated levels of oral diseases in this population [15]. In a recent study among Palestinian adolescents living in underserved areas, a strong relationship was found between the mother’s education and father’s employment status and the children's level of disease [11].

Other sociopsychological factors, such as Parental Level of Stress (PLS), Locus of Control (LOC), and Social Support (SS) have been identified as important determinants of ECC [16–20] no 19 here. These factors among other individual factors can influence diet and preventive practices as well as seeking dental care, and all these individual level factors mediated by upstream environmental and institutional determinants may change the ecology of the oral microbiome to create dysbiosis [21].

The complex and dynamic interactions among these factors and their influences on the levels of ECC are not yet fully understood. In one study [20], the social support that the mother gets from her family and friends and the level of stress (PSL) that she suffers were related significantly to their children's oral health status. In another study by McLoyd et al. [22], both social networks and PSL were identified as barriers to utilizing dental services.

There are limited data on ECC and dental caries in Palestine. However available data among older children [8, 23–25] and pregnant women [15] only found extremely high caries experience and severity in permanent teeth.

Data available on ECC prevalence in Palestine indicate high levels of the disease, as demonstrated by findings of two studies conducted in the northern areas of the West Bank governorates. In one study, 76% of 1376 children aged 4–5 years had already experienced caries, with an average dmft of 2.5 [26] In another study, 79.2% of the 450 4–5-year-old children examined had experienced caries, with an average of 4.5 dmft [27]. Both studies did not address how their dietary, social, psychological, and oral hygiene-related characteristics were related to the levels of ECC.

In this study, we hypothesize that social determinants are major risk factors for dental caries experience and severity among Palestinian children. Specifically, this study aims to evaluate the prevalence and severity of ECC among 4–5-year-old Palestinian preschoolers in Jerusalem Governorate which only include areas where Palestinians live outside the separating walls between Israeli and PA-ruled areas. The targeted population is children who are most underserved in the Occupied Palestinian Territories (oPt). The study also aims to test the association between dental caries and the caregiver’s feeding habits, the caregiver’s social and psychological characteristics, children’s oral hygiene practices, and children’s diet habits after counting for demographic characteristics and access to dental care.

## Material and methods

### Study design and sample

**Fig. 1:**
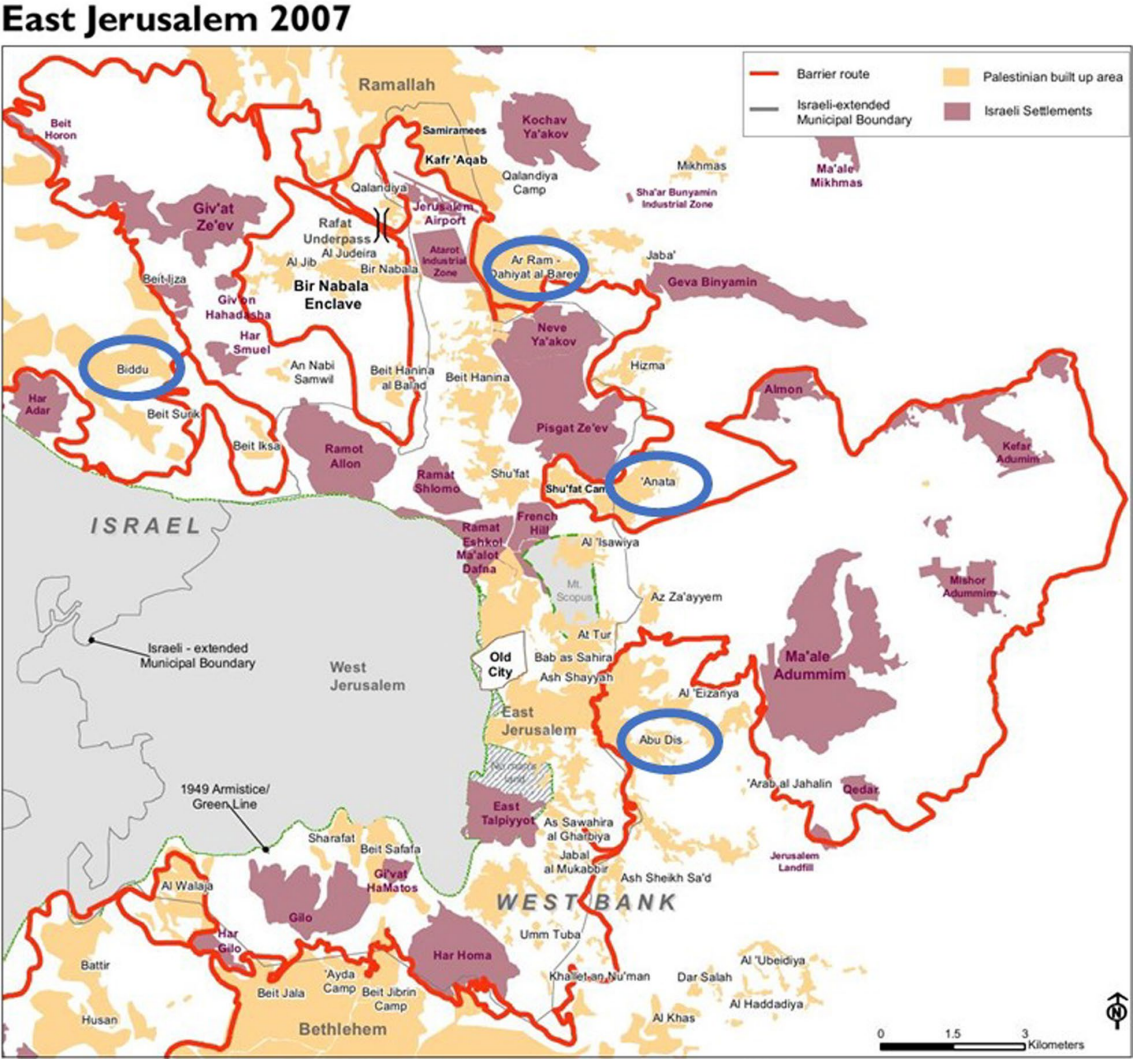
Map of preschools' geographic locations

Data for this study came from a cross-sectional survey carried out between June 2019 and January 2020 in the Jerusalem Governorate of Palestine, also referred to as the Occupied Palestinian Territories (oPt). The sampling frame included preschools registered in the Palestinian Government Ministry of Education (MOE) in the Jerusalem Governorate and located outside the Separating Wall. The separating wall built in 2002 by the Israeli side excluded Jerusalem's urban center from surrounding Palestinian villages and towns [28] (Fig. 1). The left-out towns and villages depended on Jerusalem as an urban center for health, education, recreational activities, and social services. This separation created two different healthcare systems operating inside (Israeli municipality supervision) and outside the separating wall (Palestinian Authority (PA) supervision). Therefore, the newly established Jerusalem governorates, outside the separating wall, are considered the weakest in health infrastructure among all governorates in the oPt [29].

**Fig. 2:**
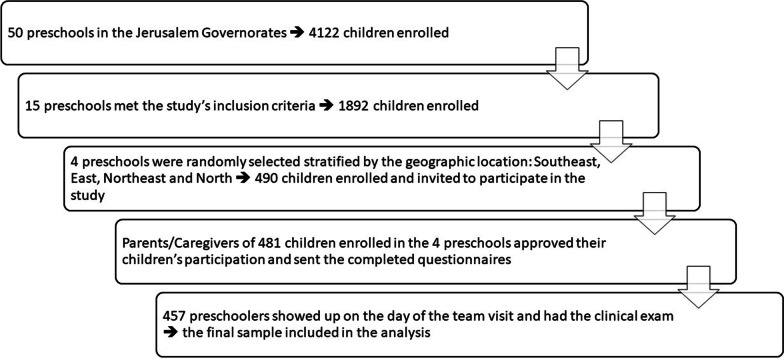
Flow chart of sample recruiting

The sampling frame of this study included preschools supervised by the PA Ministry of Education (MOE) in the Jerusalem Governorate and located outside the Separating Wall (n = 50 schools with 4122 children). Fifteen schools out of fifty met the study inclusion criteria. Inclusion criteria for the preschools were: (1) preschools located in Jerusalem governorates and situated outside the Separating Wall, (2) registered at the Palestinian Ministry of Education, (3) have both genders, and (4) have at least 100 children enrolled in the 2019/2020 academic year. A random sample of four preschools were selected and stratified based on the different geographic areas in the Jerusalem governate (North, Northeast, East and Southeast). A flow chart the demonstrate the recruitment process is presented in Fig. 2.

Based on sample size calculation, minimum of 352 subjects needed for this study. This number was based on an ECC prevalence of 50% (based on previous literature in oPt [25–27], a marginal error of 5% and a target population of (n = 4122).

All children in the selected schools (aged 3–5 years) were invited to participate in the study via letters to the parents asking for consent for their children to be examined and explaining the study's purpose and methodology. Children who were absent on the day of the examination visit and those whose parents or main caregivers did not consent to their participation were excluded from the study.

### Parents’/caregivers’ questionnaire

A self-administered questionnaire was adapted from previous studies [30, 31] and translated to Arabic, the mother tongue of the Palestinians. The Arabic version was reviewed by a panel of 6 experts from the disciplines of Pediatric Dentistry and Public Health. Pilot testing of the Arabic version of the questionnaire was carried out on a sample of fifteen mothers in a preschool in Bethlehem Governorate. Modifications were added to make the questionnaire more readable, meaningful, and culturally appropriate.

The questionnaire was sent home to the mothers or the main caregivers in the family to answer before the day of the clinical screening visit to the preschool. The mothers/main caregivers were asked about feeding practices used for the children when they were infants, current dietary habits, oral hygiene habits, and their access to dental care. In addition, demographic and socioeconomic information about the parents was collected. Socio-psychological factors among the main caregivers were also collected through validated instruments [17, 21].

### Demographic data

Demographic data included the child’s age, gender, and socio-economic status determined by parents’ level of education and family income. The child’s age was recorded as (a) 4-year-old, or (b) 5-year-old. Information regarding the level of education was requested separately for the father and mother using a five‐point scale ranging from less than eighth-grade education to 4 years of college and more. Father’s employment status (regular job, irregular job, unemployed) and the mother’s employment status (full-time job, part-time job, student, stay-at-home) were also assessed separately. The monthly household income was given in New Shekels (NIS) and ranged on a six-point scale from (1) less than 1000 NIS (285$) to (6) 4000 Nis (1700$) and more. The current residence of the family was assessed as a city, village, or camp. Children’s access to dental care was assessed by the following questions: the family has dental insurance (yes/no), the child’s last dental visit (yes/no), and the reason for the visit (preventive visit/checkup or restorative care/problem).

### Feeding habits

Questions about feeding habits covered the main method of feeding during infancy, breastfeeding duration if the mother used to breast-feed her infant for a limited number of meals or on-demand (after 6 months of age), bottle-feeding duration, the usual contents of bottle feeding, and if the mother added sugar to the bottle or use a sweetened pacifier. Another set of questions was asked about night feeding habits: such as what is done in case the child wakes up during the night, how many times mothers usually feed their child during the night, do they use to breast-feed their children at night after the age of 6 months, and if the child uses to fall asleep while breastfeeding at night.

### Children’s current dietary habits

Questions about the frequency of snacking between meals and daily soda/sweetened juices intake were measured on a 5-point scale (never, seldom, once/day, twice/day, more than three times/day). In addition, the type of snacks preferred (salty/sweet) was asked in the current questionnaire.

### Children’s oral hygiene habits

Oral Hygiene habits of the child were assessed by the following questions: (1) at which age did tooth brushing start (not yet, younger than 2 years, 2–3 years, 3–4 years, 4–5 years), (2) frequency of tooth brushing (no brushing, irregular, once/day, twice/day) and (3) if the mother or the main caregiver assists the child in tooth brushing (yes/no).

### Psycho-social questions

The questionnaire also assessed some psychosocial constructs that were measured by validated scales used in the literature that assessed ECC, namely, the Instrumental Social Support (ISS), Locus of Control (LOC), and Parental Stress Level (PSL). The PSL instrument [17] included six items scored on a Likert scale from 1 (“never”) to 5 (“almost always”). The final score of the scale was the sum of the rating of the six statements and ranged from 6 to 36. Higher scores indicated higher levels of stress.

The ISS instrument [17] comprised four items that scored “yes” or “no.” The social support instrument was calculated as a sum of the answers; each “yes” received a “2”, and each “no” received a “1”. This the ISS ranged from 4 to 8; the higher the summated result was, the more social support the mother received. The LoC scale that had been adopted by Lenčová et al. [21] was used in this study. Based on the Locus of Control theory, this scale measures the external locus of control, internal locus of control, and belief in bad luck and chance. Each item was measured on a five-point Likert multi-item scale [ranging from strongly disagree (1) to strongly agree (5)]. The coding for the negatively formulated items (items expressing more external LoC or relying on chance) was reversed so that for the overall LOC scale (ranging from 13 to 65), higher scores reflect more positive attitudes (stronger internal LoC).

### Clinical examination

Before the clinical dental examinations, three interns at the Ministry of Health were trained and their dental examinations were standardized by the study principal investigator (E.K). The standardizing session included a double examination of ten children; then an inter-examiner reliability of the diagnoses regarding the presence of decayed teeth (dt) was calculated. A kappa agreement of 0.8 and 0.85 were obtained among the three examiners. Dental examinations were carried out with the help of a headlamp and a disposable examination set containing a mirror and dental explorer. The decayed, extracted due to caries and filled teeth index (dmft) for primary teeth was used to quantify dental caries experience among preschoolers. The criteria for caries diagnoses conformed to the World Health Organization Oral Health Basic Survey recommendations [32].

Plaque accumulation was recorded using The Silness-Löe Plaque Index (PI) [33]. PI was categorized as follows: PI 0: no observable plaque; PI 1: a thin film of plaque detected at the gingival margin by running a probe or explorer across the tooth surfaces; PI 2: a moderate amount of plaque detected along the gingival margin, plaque clinically visible; PI 3: heavy plaque accumulation detected at the gingival margin and in the interdental spaces. This index was measured at 4 points on 6 teeth, then averaged as follows: < 1: excellent oral hygiene; 1–1.9: good oral hygiene: 2–2.9: fair oral hygiene; ≥ 3: poor oral hygiene [33].

### Ethical consideration

Al-Quds University Human Subject Research Ethics Committee (74/REC/2019) reviewed all study aspects. The participation was completely voluntary. Mothers or main caregivers were asked to give informed consent to participate in the study. Identifiers data of the subjects were removed from exam sheets by the PI and were entered into the database anonymously with a numerical code only.

### Statistical analysis

**Fig. 3:**
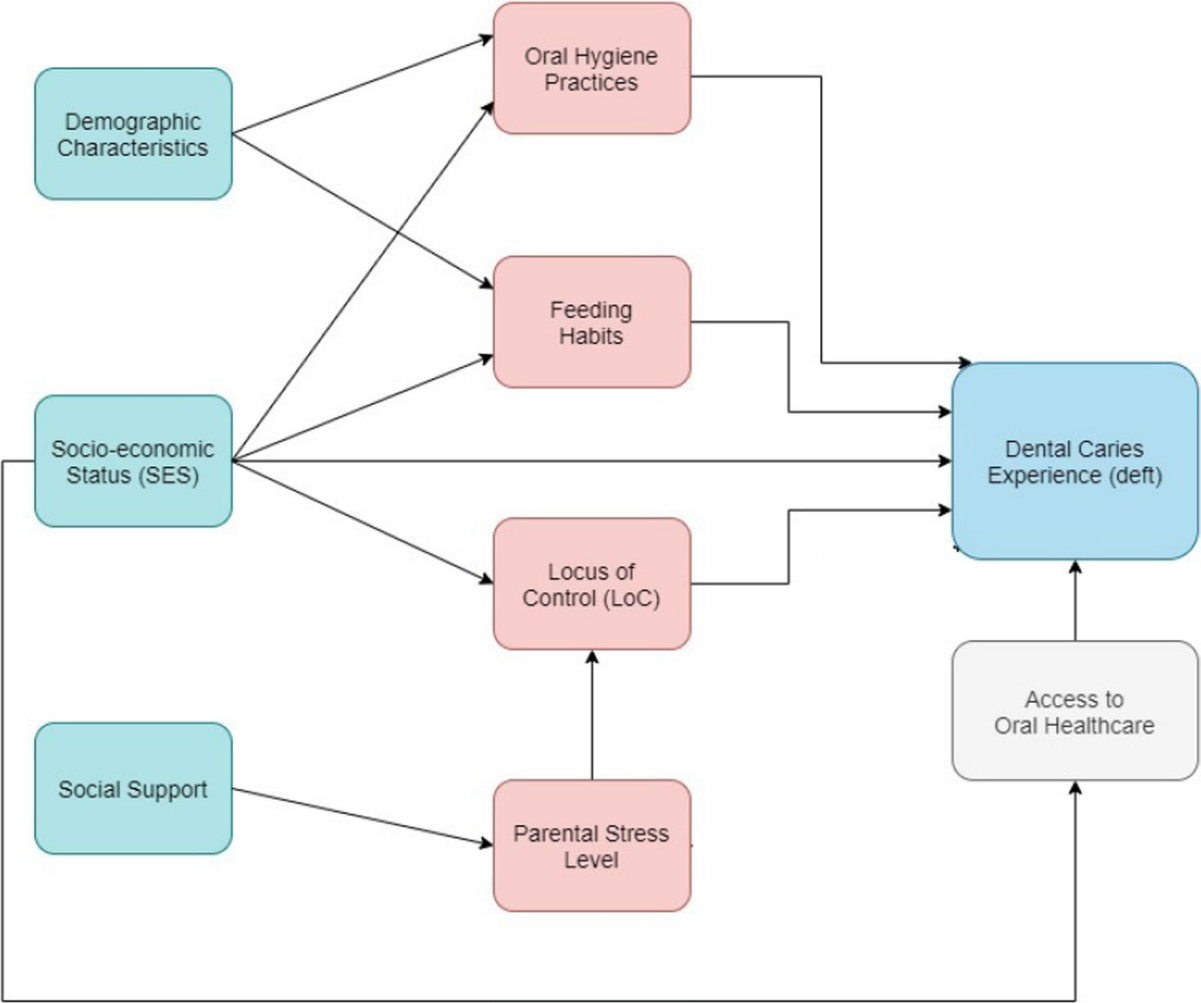
Study conceptual model

We first constructed scales to combine variables for children’s diet habits and children’s oral hygiene practices via principal component analysis. Specifically, we used the Factor Analysis of Mixed Data (FAMD), a principal component analysis for both continuous and categorical variables and identified principal components that explained 70–75% of the total variance of linear combinations of selected variables. For the scale of children’s current diet habits, we combined 3 variables (frequency of snacking between meals, type of snacks preferred, and daily soda/sweetened juices intake) into 2 scores (Children's diet habits #1 and #2) that explained 73% of the total variance of these variables. For the scale of children’s oral hygiene practice, we combined 3 variables (age at which tooth brushing started, frequency of Tooth Brushing, and helping children brush teeth) into 2 scores (Children's oral hygiene practice #1 and #2) that explained 76% of the total variance. We then calculated descriptive statistics for all study variables. In particular, for the outcome, we calculated the mean dmft (quantify the severity of dental caries), dt (untreated dental caries), and ft (dental treatment the children received) scores. The dmft score was further categorized into five levels (very mild, mild, moderate, severe, and extremely severe) according to the WHO classification of dental caries severity [32]. Lastly, we performed multivariable Poisson regression analysis to test the association between dmft scores and caregiver’s feeding habits, caregiver’s social and psychological characteristics, children’s oral hygiene practices, and children’s diet habits after accounting for demographic characteristics (caregivers’ and children’s age, children’s gender, caregivers’ education and employment, household income), dental insurance, and access to dental care, Fig. 3.

To address bias due to children clustered by schools, we used a generalized estimation equation with an exchangeable correlation structure. We also performed multiple imputations and generated 5 imputed data via multivariate imputation by chained equations. In this algorithm, missing data from one variable were predicted by regression models with all the other variables, and the imputation procedure was repeated multiple times to reduce the error of prediction of missing data [34].

Statistical significance was determined if a two-sided *p* value < 0.05. The statistical analysis was performed using SPSS version 22.0 (IBM Corp. Windows, Armonk, NY, USA), and RStudio (RStudio Team, 2020). Specifically, the R MICE package and geepack package were used for multiple imputations and regression analysis with generalized estimation equation, respectively.

## Results

Four hundred eighty-one preschoolers’ parents/caregivers completed the questionnaire, and 457 children completed the clinical screening. Children who showed up on the day of dental exam and had the completed questionnaire with the consent form were included in the current analysis (n = 457).

### Demographic and socio-economic variables

Almost 48% of our sample were females (n = 217) and 67.5% were 5-year-old (n = 307). Out of 453 answered this question, 40.4% of mothers and 64% of fathers had a high school diploma or lower. Seventy-one percent of fathers had a regular job (n = 319) and 22.4% (n = 101) of mothers worked full-time. Almost 45% (n = 189) of the preschoolers had both parents work outside the house. Thirty-eight percent (n = 162) of preschoolers lived in a household with an income equal to or less than $850 (US dollars) a month. Seventy-eight percent of preschoolers pay $720 (US dollars) or less a year as tuition for kindergarten (Table 1).

**Table 1:** Study sample characteristics

Variables	Frequency	Valid percent
*Age of the child*		
3 years	7	1.5
4 years	141	31.0
5 years	307	67.5
*Gender of the child*		
Male	237	52.1
Female	217	47.7
*Father’s education level*		
8th grade or lower	88	19.3
Lower than high school	109	23.9
Finished high school	95	20.8
Diploma—2 years or more	44	9.6
Bachelors—4 years or more	120	26.3
*Mother’s education level*		
8th grade or lower	20	4.4
Lower than high school	83	18.3
Finished high school	80	17.7
Diploma—2 years or more	62	13.7
Bachelors—4 years or more	208	45.9
*Father’s employment*		
Regular work	319	71.4
Irregular work	119	26.6
Unemployed	9	2.0
*Mother’s employment*		
Fulltime	101	22.4
Parttime	20	4.4
Student	4	0.9
Housewife	326	72.3
*Household monthly income*^a^		
Less than 285$	20	4.7
285$–570$	35	8.3
571$–856$	107	25.3
857$–1142$	102	24.1
1143$–1714$	109	25.8
1715$ and more	50	11.8
*Type of insurance that covers dental care*		
Public insurance	104	23.5
Private insurance	22	5
No insurance	317	71.6
*Has the child visited dentist before?*		
Yes	203	44.6
No	252	55.4
*Reason of dental visit*		
Routine or preventive check up	65	29.3
Treatment	157	70.7

^a^In 2017, the poverty line and the deep poverty line for a reference household of five individuals (2 adults and 3 children) were, respectively,705$ and 564$: https://www.pcbs.gov.ps/Document/pdf/txte_poverty2017.pdf?date=16_4_2018_2

### Feeding practices

Almost 45% (n = 199) of mothers in this sample reported that they exclusively breastfed their children and 11% (n = 49) used bottle feeding and 44% (n = 200) used both. Sixty-one percent of mothers (n = 279) in our sample used to breastfeed their children after 6 months of age at night and 27.7% used night bottle feeding (n = 121). Twenty-one percent (n = 84) added sugar to the bottle and 9% (n = 39) dip pacifiers in sugar or honey. Twenty-one percent (n = 81) of mothers who used bottle feeding added sugar to the bottle and 19% (n = 87) used to dip pacifiers in sugar or honey to soothe their babies.

### Current dietary habits

Fifty-three percent of preschoolers (n = 231) snack twice a day, a sweet one (80%). Thirty-one percent of children in this sample (n = 138) reported to usually have one can of soda per day or more and 69% (n = 310) never or rarely have any (Table 2).

**Table 2:** Preschoolers’ current diet habits

	Frequency	Percent (%)
*My child drinks carbonated soda*		
Never	35	7.8
Rarely	275	61
Once a day	95	21
Twice a day	35	7.8
More than 3 times a day	9	2
*My child snacks between meals*		
Never	0	0
Rarely	34	7.8
Once a day	77	17.7
Twice a day	231	53
More than 3 times a day	94	21.6

### Oral hygiene practices

Regarding daily oral hygiene care, 52% (n = 237) of children started brushing with fluoridate toothpaste after the age of 3 years and 45% of them (n = 199) brushed their teeth irregularly. Seventy-six percent of the children (n = 335) brushed their teeth under their parents’ supervision (Table 3).

**Table 3:** Oral hygiene practices among preschoolers

Oral hygiene practices	Frequency	Percent
*Frequency of your child brushing with fluoridate toothpaste*		
Not at all	44	9.9
Irregular	199	43.5
Once a day	141	30.9
Twice a day	59	12.9
*How old was your child when you started cleaning his teeth with toothbrush and toothpaste?*		
Hasn’t started yet	47	10.4
Younger than 2 years	33	7.3
2–3 years	133	29.6
3–4 years	121	26.9
4–5 years	116	25.8

In clinical examination, Plaque index (PI), which quantifies plaque accumulation, scored on average 1.02 ± 0.4 (0–3 range) and correlated positively with dmft scores, r = 0.21, *p* < 0.001.

According to Table 4, most of the preschoolers in this sample fell in the “Good category” of the Oral Hygiene classification which ranged from poor oral hygiene to excellent oral hygiene.

**Table 4:** Oral hygiene classification based on plaque index values

	Frequency	Valid percent
Excellent	128	30.5
Good	285	67.9
Fair	6	1.4
Poor	1	0.2
Total	420	100.0
*Missing*		
System	37	
Total	457	

### Access to dental care

Access to dental care was assessed by having a dental visit before. Forty-four percent (n = 203) answered yes. Seventy-one percent (n = 157) visited the dentists for therapy and 29% (n = 65) for routine and preventive care reasons. Among our sample, 72% (n = 317) paid out of pocket for their dental care, 5% (n = 22) had private insurance and 23.5% (n = 104) had public insurance.

### Socio-psychological characteristics

For psychosocial factors reported by preschoolers’ mothers or main caregivers, social support in this sample (n = 457) ranged from 4 to 8 with a mean score of 6.1 ± 1.3 and a median of 6.0 and an interquartile range of (7–5). Mothers' Level of Stress (PLS) (n = 442) ranged from 6 to 28 with a mean score of 16.4 ± 4.2 and a median score of 16 and an interquartile range of (19–13). The Locus of Control measure ranged (n = 396) from 28 to 62 with a mean score of 48.3 ± 5.2 and a median of 49.0 and an interquartile range of (52–45). For all psychological scales, higher scores indicated higher, stress levels, a sense of locus of control, and higher social support (Table 5).

**Table 5:** Social and psychological scales of preschoolers parents as reported by the mother or the main caregiver

	Parents' stress level scale	Parents' locus of control scale	Social support scale
Number of respondents	442	396	456
Missing	15	61	1
Mean	16.4	48.3	6.1
Median	16.0	49.0	6.0
Std. deviation	4.2	5.2	1.3
Minimum	6.0	28.0	4.0
Maximum	28.0	62.0	8.0
*Percentiles*			
25	13.0	45.0	5.0
50	16.0	49.0	6.0
75	19.0	52.0	7.0

Descriptive statistics of Social and Psychological scales using imputed data can be found in Additional file 1: Appendix 1

### Dental caries prevalence and severity

Among those who completed the clinical examination, 97% (n = 447) had at least one dmft, with an average score of dmft score of 6.6 ± 4. The main component of dmft scores was untreated dental decay (dt score of 6.2 ± 4.2) and filling (ft score of 0.34 ± 1.1). The severity of the dental experience was quantified by a dmfs score of 14.0 ± 13. A detailed description of preschoolers' dmft scores according to the WHO “Very Low” to “Extremely High” categories is shown in Table 6.

**Table 6:** dmft classification among the sample’s preschoolers according to the WHO

Dental caries experience	Frequency	Percent
Very low	54	11.8
Low	36	7.9
Moderate	69	15.1
High	83	18.2
Extremely high	215	47.0
Total	457	100.0

### Bivariate and multivariable analyses

Bivariate associations between the prevalence of dental caries (dmft score) and study main exposure variables were tested (Fig. 3). Behavioral and psychological factors were significantly associated with the dmft scores (Table 7). Specifically, putting things other than water in the baby bottle and sleeping while naturally breastfeeding were related to higher dmft scores (IRR = 1.05, 95% CI = 1.01, 1.10, IRR = 1.14: 95% CI = 1.03, 1.25, respectively). On the other hand, not adding sugar to the bottle was associated with lower dmft scores (IRR = 0.80, 95% CI = 0.69, 0.92). Access to dental care was a significant factor in dmft scores. Having routine, preventive visits versus never seeing a dentist were associated with lower dmft scores (IRR = 0.44, 95% CI = 0.38, 0.51). For psychological factors, parents’ internal locus of control was associated with lower dmft scores (IRR = 0.97, 95% CI = 0.96, 0.98). Although PSL was not directly associated with dmft scores, it was correlated with LOC (r = − 0.133, *p* = 0.012) and SS (r = − 0.16, *p* = 0.001).

**Table 7:** Results from bivariate and multivariable regression analyses

Predictor variables	Bivariate analysis			Multivariable analysis		
	IRR	95% CI		IRR	95% CI	
Locus of control scale	0.97	0.96	0.98	0.97	0.97	0.98
Parental stress scale	1.00	0.99	1.02	1.00	0.98	1.01
Social support scale	0.99	0.96	1.03	0.99	0.99	1.02
What you do when your baby wakes up in the night? Baby bottle with things other than water (yes vs. no)	1.05	1.01	1.10	1.06	1.04	1.09
Does your child normally sleep while naturally breastfeeding? (yes vs. no)	1.14	1.03	1.25	1.15	1.03	1.29
How long did you depend on artificial feeding? (limited number of meals set by mother vs. whenever the child asked)	0.98	0.91	1.06	0.95	0.87	1.02
Adding sugar to the bottle (no vs. yes)	0.80	0.69	0.92	0.86	0.74	1.00
What do you used to put in the baby bottle? (juices)	1.14	0.89	1.47	0.99	0.76	1.29
What do you used to put in the baby bottle? (dry milk)	0.97	0.88	1.06	0.94	0.87	1.01
Length of natural breastfeeding (none or < 3 months vs. 3 months–2 years)	1.09	0.92	1.30	1.11	0.95	1.30
Length of natural breastfeeding (more than 2 years vs. 3 months–2 years)	1.21	0.95	1.53	1.28	0.88	1.87
What determined breastfeeding after 6 months?	1.09	0.94	1.26	1.08	0.91	1.29
Scale 1: Children's diet habits #1	1.15	1.04	1.27	1.04	0.97	1.12
Scale 1: Children's diet habits #2	0.90	0.66	1.22	0.92	0.73	1.14
Scale 2: Children's oral hygiene practice #1	1.10	0.97	1.25	1.06	0.88	1.27
Scale 2: Children's oral hygiene practice #2	1.00	0.68	1.46	0.99	0.73	1.33
Routine or preventive check-up versus no visit	0.44	0.38	0.51	0.42	0.33	0.52
Treatment visit versus no visit	0.72	0.52	1.01	0.68	0.44	1.04

Multivariable Poisson regression with generalized estimation equation included the following covariates to account for potential confounding: age of the child, gender of the child, father’s education level, mother’s education level, father’s employment, mother’s employment, household monthly income, and insurance type

After accounting for potential confounding, parents’ internal locus of control was associated with lower dental caries among children (IRR = 0.97, 95% CI = 0.97, 0.98). When it comes to the burden of nighttime feeding, a higher burden (putting things other than water in the baby bottle at night, having children sleep while being breastfed at night) was positively associated with children’s dental caries (IRR = 1.06, 95% CI = 1.04, 1.09: IRR = 1.15, 95% CI = 1.03, 1.29, respectively). Not adding sugar to the bottle was negatively associated with children’s dental caries (IRR = 0.86, 95% CI = 0.74, 1.00). Lastly, after accounting for potential confounding, “having routine, preventive visits” versus “never seeing a dentist” was associated with lower dmft scores (IRR = 0.42, 95% CI = 0.33, 0.52). Bivariate and multivariable analyses can be found in Table 7.

## Discussion

ECC is a strong predictor of dental caries in mixed and permanent dentition and often persists into adulthood [35]. Children in this sample suffer from a high prevalence of untreated dental caries. Social determinants of health including psychological, economic, and behavioral factors were detrimental factors in explaining the high level of diseases. According to the WHO, ECC was prevalent in 30% of Africa, 48% in the Americas, 52% in Asia, 43% in Europe, and 82% in Oceania [36]. Considering the 48% global ECC prevalence, the prevalence of 97% ECC found in our study is extremely high. Those preschoolers had a dmft mean of 6.6, placing 65% of them in the “high” and “extremely high” caries experience category according to the WHO classification. In a systematic review [8] that summarized results from 77 articles published from 2000 to 2019 and included 94,491 participants in 14 countries across the MENA (Middle East and North Africa) region, the ECC prevalence ranged between 3 and 57%, and the dmft average varied between 0.6 and 8.5 across different age groups. The dmft scores in our study were clearly in the higher bracket of all MENA region.

In the previous systematic review, low maternal education and low overall socioeconomic status were among the main drivers of the high level of the disease. This agrees with the current study results that parents’ education level and household income influenced dmft scores through their influence on the parental LOC levels where higher education levels and household incomes increased the LOC among parents in this sample. Parental stress was associated with higher dental caries in a low-income community in Detroit [17, 18]. Although parental stress was not associated with dental caries in this study, we believe that it might be indirectly associated with dental caries given that parental stress was highly correlated with LOC. We found that higher parental LOC was significantly associated with lower dental caries among preschool children, and this finding was consistent with the previous literature [21, 37] that identified LOC as an important factor in dental caries experience where a positive effect of strong parental LoC was found on the level of untreated caries of preschool children [21]. Given that parental LoC was highly correlated with parents’ socio-economic conditions, our findings suggest that improving the socio-economic conditions could empower parents to take more control of their children's oral health and minimize the level of the disease which emphasizes the concept of the crucial influence of social determinants on oral health.

The results of this study agree with the 14 MENA countries' data, where infant feeding practices and sugar consumption were among the most prevalent determinants for increased risk of dental caries. Unfavorable feeding habits such as using the bottle for sugary liquids, adding sugar to the milk in the bottle feeding, and children falling asleep while breastfeeding was associated with high caries experience in the current study. This finding should be used to design an oral health promotion campaign to encourage healthy feeding habits (Additional file 1).

In the MENA region data, poor oral hygiene habits and low brushing frequency were also associated with high levels of disease. In Palestine, most of toothpastes available for sale are fluoridated; however, in our study, the late introduction of fluoride by starting brushing after the age of 3 was found among 52% of the sample. This, along with the poor feeding and diet habits, could explain the elevated level of the disease among our sample. In this sample and the Palestinian territories in general, toothpaste seems the only source of fluoride exposure given that professional fluorides are not a widespread practice and the fluoride levels in drinking water in the West Bank areas are below optimum [38]. This is another area where support is needed from the Government and other Non-Governmental Organizations to promote the application of fluoride varnishes as an integral service with other health initiatives such as vaccinations.

The study sample was only drawn from the Jerusalem Governorate and schools located outside the Separating Wall. This makes our sample unique due to the geopolitical status quo of this area and generalizability to other Palestinian governorates should be taken with caution. Including only children who are enrolled in preschools (preschool education is not mandatory in oPt) may also affect the generalizability of our results. In addition, some of the data were collected retrospectively (data about feeding practices) which may cause some response bias. This study also has the limitation of cross-sectional research design in general which limits the use of bidirectional data in inferring causality between risk determinants and levels of disease.

A more comprehensive national survey with a sample representative of preschoolers in all oPt governorates is needed to fully assess the burden of ECC among Palestinian children. Despite the limitations, the current study’s results provide good basis to advocate for oral health policy changes and interventions planning and implementation among this age group.

The majority of our sample are under the poverty line and do not have insurance to cover basic dental care. All this made access to dental care extremely challenging in this sample where families take their children to see a dentist mainly when they are in pain. Access to proper care can raise awareness among parents of the essential basics of oral health self-care and increase the chances of exposure to professional fluoride therapy. Therefore, lack of access to care can explain the high level of untreated dental caries compared to children in the same age group in two governorates in the north of the West Bank, Jenin (76% prevalence with a 4.5 dmft) [23] and Nablus (79% prevalence with a 2.5 dmft) [24].

The recent WHO strategy for Oral Health recommended placing equity and social justice at the core, to address the social determinants of oral diseases and embrace major system reforms [39]. The results of the current study suggest that better access to dental care should be prioritized among this underserved population by addressing social determinants of health in the special geopolitical context of the Jerusalem Governorate. In addition, tailored interventions should be implemented to increase awareness about feeding and diet unfavorable practices and to empower parents to take control of their children’s oral health.

Preschoolers in the study sample suffered from extremely high dental caries experience. Although infant feeding habits, current diet habits, and oral hygiene practices are important factors in explaining the high level of the disease, socioeconomic determinants, and psychological factors were, directly and indirectly, related to the ECC burden. Addressing only behavioral and biological factors in the attempts to prevent ECC is inadequate. Policies and interventions to alleviate the burden of ECC need to address socioeconomic determinants of health and be integrated into programs aiming to prevent other NCDs that share the same common risk factors with ECC.

## Supplementary Information

**Additional file 1**. **Appendix 1.** Descriptive statistics of Social and Psychological scales using imputed data.

## Data Availability

The datasets used and analyzed during the current study are available from the corresponding author upon reasonable request.

## Abbreviations

ECC: Early childhood caries
dmft: Decayed, missing and filled teeth for primary dentition
dmfs: Decayed, missing and filled surfaces for primary dentition
WHO: World Health Organization
ISS: Instrumental social support
LOC: Locus of control
PSL: Parental stress level
