# Supplementary material for: Behavioral and social determinants of early childhood caries among Palestinian preschoolers in Jerusalem area: a cross-sectional study
Source: BMC Oral Health. 2023 Mar 15;23:152. doi: 10.1186/s12903-023-02809-2 (PMC10015793; doi:10.1186/s12903-023-02809-2)
Supplement: Supplementary file 1 — Additional file 1. Appendix 1. Descriptive statistics of Social and Psychological scales using imputed data. [file 12903_2023_2809_MOESM1_ESM.docx]

Appendix 1. Descriptive statistics of Social and Psychological scales using imputed data

|  | Mean | Standard error |
| --- | --- | --- |
| Parents’ stress level scale | 16.4 | 0.2 |
| Parents’ locus of control scale | 48.2 | 0.3 |
| Social support scale | 6.1 | 0.1 |
